# The Relationship Between Chinese College Student Offspring's Physical Activity and Father Physical Activity During COVID-19 Pandemic

**DOI:** 10.3389/fpubh.2022.896087

**Published:** 2022-06-03

**Authors:** Feng Zhang, Wei Liu, Lamei Deng, Zhifu Li, Yajun Wang, Bo Jin

**Affiliations:** ^1^Physical Education Institute, Xuzhou University of Technology, Xuzhou, China; ^2^Department of Physical Education, Anhui Vocational and Technical College of Sports, Hefei, China; ^3^Students' Affairs Division, Hangzhou Medical College, Hangzhou, China; ^4^Department of Electrical and Computer Engineering (DEEC), Institute of Systems and Robotics (ISR), University of Coimbra, Coimbra, Portugal

**Keywords:** physical activity, retroactive socialization, parents, intergenerational, empirical study

## Abstract

The purpose of this study was to evaluate the relationship between the physical activity of Chinese college students and the physical activity of their parents. This relationship was examined by linear regression. The results showed that (1) the physical activities of China's college students will be refurbished and used for the parents. The offspring of COVID-19's later generation sports population showed that the offspring's physical activity was not broken during the later stage of the epidemic, and the coefficient of promotion (*r* = 1.515) (*p* < 0.01) of the offspring's participation in the physical activities was. (2) The increase or decrease of parents' sports population is affected by their children's occupation. Therefore, it shows the dynamic role of individuals and the two-way nature of socialization in the process of socialization. With the transformation from traditional society to modern society, promoting college students' physical activities can increase parents' physical activities and improve the level of social physical activities.

## Introduction

Retroactive socialization was first proposed by Riesman and Roseborough in 1955. It mainly refers to the inter-generational influence of the younger generation on the parents' generation. Usually, the family intergenerational interaction studies the offspring centered on the parent-child capital. However, with the development of society, the two-way interaction between generations is significantly strengthened. As the subject of socialization, parents need to be socialized by their corresponding due objects. At present, the domestic research on retroactive socialization mainly focuses on three points: first, cultural back-feeding: the cultural absorption process from the older generation to the younger generation under the background of rapid cultural change (1); second, the process of socialization of young people to the previous generation from the perspective of sociology; and third, the “post metaphor culture” in Margaret Mead's cultural studies, that is, the youth model culture in which the older generation learns from the younger generation.

During the COVID-19 pandemic, the China Sina microblog found the topic of “how to graciously persuade older generations to wear masks” and “prevention of family gatherings.” These topics are reflected in some aspects of reverse socialization. Retroactive socialization has changed the relative position between generations to a certain extent. Liu (2) believes that in the field of cultural life, the daily behavior of children has an important impact on their parents. Li (3) found in a survey that the reverse transmission from children to parents in the Internet age is not only the knowledge and skills but also the lifestyle and values. Li (4) found that it is a common phenomenon that parents are influenced by their children in their daily behavior through the study of their parents. Sports, as a form of lifestyle, can be considered that the sports participation of children will affect the possibility of sports participation of parents. In this regard, Hyatt (5) found evidence of retroactive socialization among the parents of 15 sports fans through interviews with the parents of 20 sports fans.

The theoretical and empirical studies of retroactive socialization are based on the analysis of social practical phenomena, although there is a lack of theoretical or empirical research on the impact of retroactive socialization on sports participation. However, it is undeniable that the theory of retroactive socialization confirms our life experience; that sports participation is an important method of parent-child interaction between parents and children. Therefore, regression analysis based on simple descriptive statistics and text analysis is still of great theoretical significance to explore the increase and decrease of retroactive socialization in the sports population of parents.

## Method

### Participants and Procedure

The individual data of this paper come from the data distributed by the research group to 7 colleges and universities in the Yangtze River Delta region of China, namely, Shanghai, Jiangsu, Zhejiang, and Anhui. A total of 1,946 questionnaires were collected. Because the online questionnaire cannot face the respondents, to improve the accuracy of the questionnaire, the questionnaire filling time was <40 s, the missing data and invalid questionnaires were deleted, and, finally, a total of 1,788 samples were included in the analysis.

## Measures

### Dependent Variables: The Sports Population of Parents Increased in the Late Stage of the Epidemic, and the Sports Population of Parents Decreased in the Late-Stage

“Increase in physical population of parents in the later stage of the epidemic” and “decrease in physical population of parents in the later stage of the epidemic” are measured by the frequency of physical exercise of your parents during the epidemic. The answers to this question are divided into four categories: exercise every day = 1, more than three times a week = 2, exercise occasionally = 3, and never exercise = 4. In response to the related concept of sports population, this paper defines sports population = 1 for options 1 and 2, and non-sports population = 0 for options 3 and 4.

### Independent Variable: Town + Social Status + Offspring

Based on the theory of reverse socialization, this study mainly discusses the influence mechanism of the increase and decrease in the sports population of parents during the epidemic period and selects cities and towns, social status, and the situation of a college student as independent variables, mainly for the analysis of exclusion.

Cities and towns are mainly the residences of respondents. China's urban-rural dual system structure and degree of marketization lead to sound urban sports facilities, and the per capita share is greater than that of rural areas. There are obvious differences in mass sports participation between urban and rural areas (6). Considering that urban factors may cause an increase or decrease in the sports population, this paper directly uses Urban = 1, and rural = 2 as the measurement index, so residence is included in the independent variable index.

From the perspective of sociology, the behavior of those participating in sports has obvious characteristics of social stratification. According to Bourdieu's perspective framework of social and cultural strata, mainly includes social capital, economic capital, and cultural capital. Here, the measurement of the three types of capital in the parent generation refers to Eriksson's method; that is, the husband and wife adopt the standard of the higher one for class judgment and comparison (7). The usual method of social capital is the career orientation method (8). Considering that the respondents are college students and are online questionnaires, they cannot be supervised on-site to fill in according to the occupational classification, so the parental career options in the questionnaire are divided into five categories: farming and working = 1, nature of enterprise = 2, a civil servant of public institution = 3, business = 4 and others = 5. Economic capital generally includes family income and wealth. This paper uses the annual family income as the strategic index: <100,000 yuan = 1, 100,000 to 200,000 yuan = 2, and more than 200,000 yuan = 3. In general, cultural capital mostly uses the degree of education as the measurement index. The index in the questionnaire is educational background. In the study, education is transformed into the number of years of education: junior middle school and below = 9, senior high school (technical secondary school) = 12, college = 15, undergraduate = 16, and graduate = 19.

The core of this study is the retroactive socialization impact of the offspring on the parent; whether the sports population of the offspring has a significant impact if the parent becomes the sports population or no longer becomes the sports population during the epidemic considering the current situation of school sports, the students in this paper are not defined as the sports population of course. Therefore, the frequency of college students (respondents) participating in physical exercise during the epidemic period was transformed according to the sports population standard: sports population = 1 and non-sports population = 0 (Table 1).

**Table 1:** Variable descriptive statistics.

**Variable**	**Frequency**	**Mean**	**Variance**	**Min**	**Max**
Area	1,788	1.584	0.493	1	2
Incoming	1,788	1.311	0.561	1	3
Impact of the epidemic on family income	1,788	2.204	0.606	1	3
Parent's highest occupation	1,788	2.185	1.587	1	5
Maximum years of education of parents	1,788	10.253	2.18	9	19
College student's preference for sports programs	1,788	2.874	0.963	1	5
The epidemic has increased the sports population of parents	1,788	0.081	0.273	0	1
The epidemic reduces the sports population of parents	1,788	0.118	0.323	0	1
Student sports population after the epidemic	1,788	0.37	0.483	0	1
Pre epidemic parental sports population	1,788	0.362	0.481	0	1
Parental sports population after epidemic	1,788	0.325	0.469	0	1

### Reliability and Validity Test

First, the reliability and validity of the 12 scales of the data were tested by SPSS 19.0 (Table 2). Similar methods are often used in computational sociology (9–11).

**Table 2:** Reliability test.

**Frequency**	**Scales**	**Cronbach.α**
1,788	12	0.89

From the results in Tables 2, 3, the sample data of the questionnaire have good reliability (α value exceeds 0.8). Additionally, the sample results have a certain degree of authenticity (the overall value exceeds.8). Due to the high KMO value, which is much >0.8, and the *p*-value of Bartlett's sphere test is < 0.05, the validity of the scale is good.

**Table 3:** Validity test.

KMO value		0.821
Bartlett's sphericity test	χ^2^ value	33919.211
	*P-*value	0.001

## Main Result

The regression model verified that the offspring's preference for physical activities would be inversely socialized for the parents (Table 4). The variable of the physical population of the offspring in the later stage of the epidemic shows that the offspring have not interrupted physical exercise in the later stage of the epidemic. This variable is significant at *P* < 0.001 and reflects that the proportion difference between the parent and the offspring in the sports population or the non-sports population is up to 4.5 times. Model 2 is the model of reducing the sports population of parents in the later stage of the epidemic. Through the regression equation, it can be seen that the occupation of parents and the sports population of a college student in the later stage of the epidemic have a significant impact. Among them, the occupation of parents was only one of them, which was significant at *P* < 0.05, while the sports population of college students in the later stage of the epidemic was significant and negatively correlated at *P* < 0.001.

**Table 4:** Logit regression model of parental sports population changes during new coronary pneumonia.

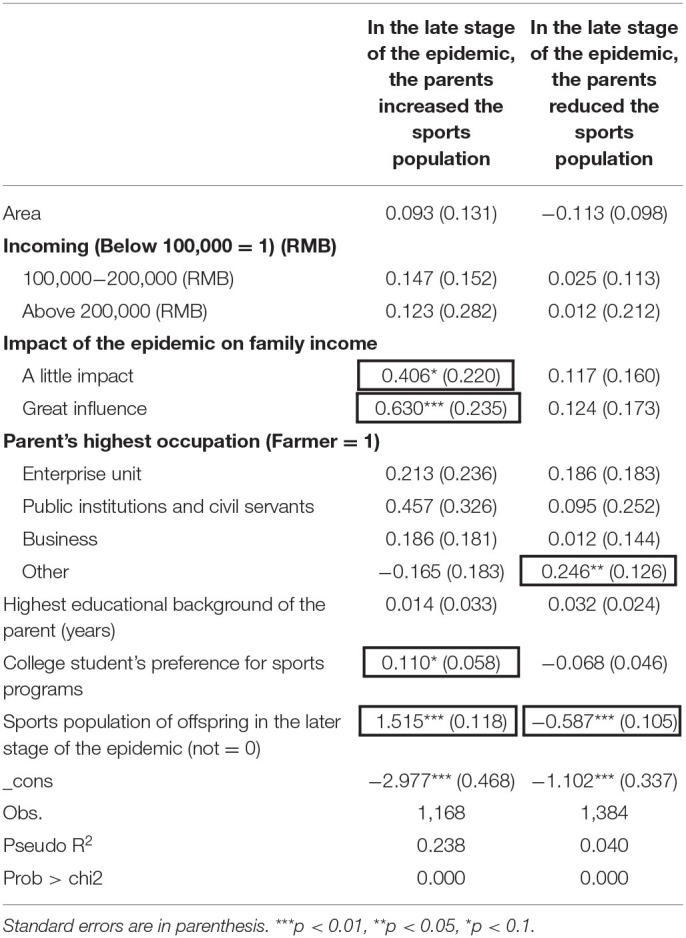

## Discussion

This part will focus on the influencing factors of the change in the sports population of the parents during the period of new-type coronary pneumonia. Through the method of counterevidence, it is deduced that the change in the sports population of the parents is not affected by the culture of the social stratum, but by the influence of the college student, which proves the increasing and decreasing effect of retroactive socialization on the sports population of the parents. Model 1 is that the parent generation increases the sports population in the later stage of the epidemic. Through the regression equation, it can be seen that there are three significant variables: the impact of the epidemic on family income, the preference of the offspring for sports programs, and the sports population of the offspring in the later stage of the epidemic. Urbanization, economic capital, social capital, and cultural capital have no significant impact on the sports population of the parent generation during the epidemic period; that is, the increase or decrease in the sports population of the parent generation is not related to its basic social attributes. The impact of the epidemic on family income has a positive correlation with the increase in the sports population of parents, and the greater the impact income is, the greater the sports population of parents, indicating that most of these groups are freelance or business, and the usual time limit makes them unable to engage in physical exercise. At the same time, the offspring's preference for sports programs is also positively correlated with the parent's sports population, which also verifies the retroactive socialization of the offspring's preference for the parent. The sports population variable of the offspring in the later stage of the epidemic shows that the physical exercise of the offspring was not interrupted in the later stage of the epidemic. Model 2 is the model of reducing the sports population of parents in the later stage of the epidemic. Through the regression equation, it can be seen that the occupation of parents and the sports population of a college student in the later stage of the epidemic have a significant impact. The coefficients of the two regression models are significant in whether the offspring is a sports population variable in the later stage of the epidemic, indicating that the increase and decrease in the sports population of the parents are affected by the offspring, which also verifies the impact of the values and life attitudes of the college student of retroactive socialization on their parents' lives.

What is the significance of discussing the increase and decrease in the sports population of parents and the retroactive socialization of college students during the epidemic? The connotation of retroactive socialization gives us three perspectives: first, the theory of cultural pioneer, that is, in the migration of social and economic life, the young generation has been the pioneer of cultural reform since ancient times, and the cultivation of physical culture in the family will affect the physical exercise behavior of the father generation; second, the direct factor theory, that is, the younger generation is more likely to accept new things, new life concepts, and new trends of thought. Youth culture has played an important role in today's social life. It can be predicted that in the future, the scientific exercise of the child generation of the family will promote the formation of the scientific exercise habit of the parent group. Third, the positive function says that the subculture of youth groups has a guiding function for the promotion of social culture (12), which can promote the progress of social sports culture through the popularization and sublimation of sports culture by offspring and contribute to the promotion and implementation of Health 2030 and national fitness plans. The regression equation model of this study also illustrates another problem, which is the relationship of mutual expectation between the sports population of parents and college students, and it is through this mutual expectation that retroactive socialization can take place. In this parent-child relationship between parents and college students, both parents and college students can reap the health and psychological satisfaction brought by physical exercise. At the same time, this retroactive socialization also plays a positive role in maintaining intergenerational relations.

Therefore, when promoting the strategy of healthy China and sports power, we should not only do a good job in social promotion, but also do a good job in the teaching reform of school physical education. In particular, we should establish the teaching reform concept of “people-oriented and health first” and actively promote the enthusiasm of parents to participate in sports from the perspective of promoting students' participation in sports.

## Conclusion

Based on the theory of reverse socialization, this study empirically analyzed the reverse intergenerational mechanism of the sports population during the epidemic transmission of new coronary pneumonia through descriptive statistics of survey data and logit regression models. Specifically, the main findings and conclusions are as follows: (1) during the epidemic period, the physical exercise frequency and physical population of parents in the family decreased slightly; (2) during the epidemic period, there was no significant correlation between the increase or decrease in the sports population and its basic social attributes; and (3) during the epidemic period, the offspring played a retroactive socialization role in the increase in the parent's sports population; the college student's physical exercise behavior affected the parent's physical exercise behavior. Whether the college student is a sports population or not has 4.5 times the impact on the parents becoming a sports population. (4) During the epidemic period, the decrease in the sports population of parents is highly related to the non-sports population of their college students. Among them, the probability of reducing the sports population is doubled if the college student is a non-sports population. In other words, through the analysis of the survey data during the epidemic period, the frequency of physical exercise of the parents in the family was lower than that of the sports population. Whether increasing or decreasing, one of the important internal factors is the promotion of offspring.

## Research Advantages and Disadvantages

The research data in this paper come from survey data during the COVID-19 epidemic. The data environment is true, reliable, and effective, verifying the correctness of the theory of reverse socialization and finding that the physical mechanism of the offspring can effectively promote the social mechanism of the parent's physical activity. However, due to the limitation of the questionnaire, the survey object of this paper is only college students, and there is a lack of data on the physical activities of middle school students and primary school students. At the same time, it is also necessary to add the relevant variables of fathers' social capital in the follow-up research to promote the research results to be more objective and effective.

## Data Availability Statement

The raw data supporting the conclusions of this article will be made available by the authors, without undue reservation.

## Author Contributions

FZ and WL drafted the work and revised it critically for important intellectual content. LD, BJ, ZL, and YW contributed to the initial drafting of the manuscript. All authors contributed to the article and approved the submitted version.

## Funding

This work was supported by a grant from the Natural and Social Science Project of Colleges and Universities in Anhui Province (No. KJ2020A1223) and the Humanities and Social Science Project of Colleges and Universities in Anhui Province (No. SK2021A1043).

## Conflict of Interest

The authors declare that the research was conducted in the absence of any commercial or financial relationships that could be construed as a potential conflict of interest.

## Publisher's Note

All claims expressed in this article are solely those of the authors and do not necessarily represent those of their affiliated organizations, or those of the publisher, the editors and the reviewers. Any product that may be evaluated in this article, or claim that may be made by its manufacturer, is not guaranteed or endorsed by the publisher.
